# Socio-Ecological Intervention to Promote Active Commuting to Work: Protocol and Baseline Findings of a Cluster Randomized Controlled Trial in Finland

**DOI:** 10.3390/ijerph14101257

**Published:** 2017-10-20

**Authors:** Minna Aittasalo, Johanna Tiilikainen, Kari Tokola, Timo Seimelä, Satu-Maaria Sarjala, Pasi Metsäpuro, Ari Hynynen, Jaana Suni, Harri Sievänen, Henri Vähä-Ypyä, Kalle Vaismaa, Olli Vakkala, Charlie Foster, Sylvia Titze, Tommi Vasankari

**Affiliations:** 1 UKK Institute for Health Promotion Research, P.O. Box 30, 33501 Tampere, Finland; johanna.tiilikainen@uta.fi (J.T.); kari.tokola@uta.fi (K.T.); jaana.h.suni@uta.fi (J.S.); harri.sievanen@uta.fi (H.S.); henri.vaha-ypya@uta.fi (H.V.-Y.); tommi.vasankari@uta.fi (T.V.); 2 Department of Transport and Streets, City of Tampere, P.O. Box 487, 33101 Tampere, Finland; timo.seimela@tampere.fi; 3 School of Architecture, Tampere University of Technology, P.O. Box 600, 33101 Tampere, Finland; satu.sarjala@tut.fi (S.-M.S.); ari.hynynen@tut.fi (A.H.); 4 WSP Finland Ltd., Kelloportinkatu 1 D, 33100 Tampere, Finland; pasi.metsapuro@wspgroup.fi (P.M.); kalle.vaismaa@wspgroup.fi (K.V.); 5 Ecofellows Ltd., Valssipadonraitti 3, 33100 Tampere, Finland; olli.vakkala@tampere.fi; 6 Centre for Exercise Nutrition and Health Sciences, School for Policy Studies, Faculty of Social Sciences and Law, University of Bristol, 8 Priory Road, Bristol BS8 1TZ, UK; charlie.foster@bristol.ac.uk; 7 Institute of Sport Science, University of Graz, Mozartgasse 14, 8010 Graz, Austria; sylvia.titze@uni-graz.at

**Keywords:** walking, cycling, workplace, health promotion, environment, multilevel, intervention, protocol

## Abstract

Active commuting to work (ACW) is beneficial to traffic, environment and population health. More evidence is needed on effective ways to promote ACW. This paper describes the protocol and baseline findings of a cluster-randomized controlled study, which aims to promote ACW with multilevel strategies in two large workplace areas in Tampere, Finland. In Phase 1, the impacts of environmental strategies (trail improvements) were evaluated in 11 workplaces within Area 1. In Phase 2, five more workplaces were recruited from Area 2 to evaluate the impacts of social and behavioral strategies customized for each workplace. For this purpose, the workplaces in both areas were randomly assigned into experimental group (EXP, n = 6 + 2), which promoted ACW with social and behavioral strategies or into comparison group (COM, n = 5 + 3), which participated in the data collection only. The primary indicator in both phases is the change in employees’ ACW. Secondary indicators are e.g., changes in employees’ self-rated health, wellbeing at work, restrictions to and motivation for ACW, adverse effects due to ACW and the use and quality of the main walking and cycling trails. Also process, efficiency and environmental evaluation is included. The study is the first one in Finland to combine interdisciplinary collaboration between practitioners and researchers working in the fields of transportation, urban design, physical activity and sustainable development to promote ACW. The findings benefit all stakeholders interested in promoting ACW in urban context.

## 1. Introduction

The prevalence of overweight and obesity is rising globally [1]. It is connected to the increased risk of cardiovascular diseases [2] and type 2 diabetes [3] and furthermore to extensive public health costs [4]. The high rate of obesity and non-communicable diseases can partly be explained by low level of physical activity [5], which has also been identified as the fourth leading risk factor for mortality [6]. 

Active commuting to work (ACW) by walking or cycling imperceptibly increases the total amount of physical activity [7,8] affecting favorably to both physical [9,10,11,12] and mental health [13,14]. Active travel enhances health and wellbeing also through reducing the risk exposures to traffic, noise and air pollution [9,15]. In addition, active travel as a form of physical activity is accessible to most people [16]—to those too, who do not have money, time, place or interest for recreational exercise. 

Infrastructure is a fundamental part of promoting active travel [17,18] and ACW [19,20]. However, studies show that also individual, social and organizational factors play an important role in adults’ ACW [21,22,23,24]. In this perspective, strategies are needed at multiple levels of peoples’ lives to change their ACW-related behavior. This calls for interventions following multifaceted approaches such as socio-ecological models [25,26,27]. 

Socio-ecological (SE) models have been successfully adopted in studies promoting physical activity in various contexts [28]. However, based on the reviews on the effectiveness [29] and frameworks [30] of active travel interventions in adults, it seems that multilevel approaches have not been widely utilized in studies promoting active travel or ACW. This paper describes the protocol, evaluation and baseline findings of the study aiming to promote ACW through environmental, social and behavioral strategies in two urban areas including a total of 16 workplaces. 

## 2. Materials and Methods

### 2.1. Ethics

The study was conducted in accordance with the Declaration of Helsinki, and the protocol was approved by the Ethics Committee of the Tampere Region, under the auspices of University of Tampere (http://www2.uta.fi/en/research/tutkimuksen-etiikka/ethics-committee-tampere-region, running number 20/2014). The participants gave their consent to participate in the study by agreeing to complete the measurements after being fully informed in writing about the ethical principles. Written informed consent was not obtained because no sensitive data were collected and the study did not intervene physical integrity of the participants. The study has been registered to the database of ClinicalTrials.gov (NCT02250261, date 09/23/2014). 

### 2.2. Participants

#### 2.2.1. Workplaces

**Figure 1:**
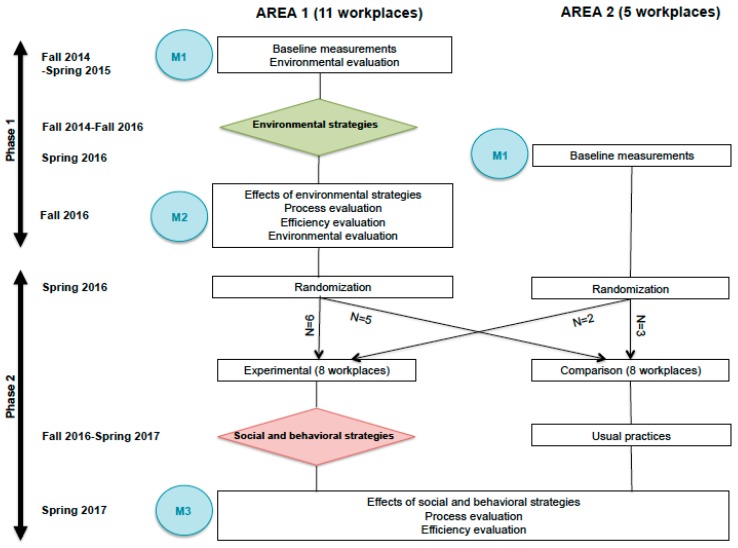
Schematic diagram of the study design including the timing of the measurements and intervention strategies. M1 = Baseline measurements, M2 = Measurements for evaluating the effects of environmental strategies, M3 = Measurements for evaluating the effects of social and behavioral strategies.

The workplaces for the study were recruited from two large workplace areas located just outside the city center of Tampere, Finland. In Area 1, the recruitment of the workplaces started in September 2014 and ended in February 2015. Area 1 was selected because the City of Tampere planned to conduct several improvements to the walking and cycling trails connected to the area. The primary aim of Phase 1 was to assess the effects of these environmental strategies on the ACW of the employees working in the recruited workplaces (Figure 1). 

The search of the potential workplaces was limited to workplaces with a minimum of 10 employees to ensure that there would still be participants left in each workplace for the follow-ups after the dropout. In Area 1 the search was made from the database of Statistics Finland. After gaining the information, it was checked with Internet searches, visits to the area and telephone calls to confirm that the workplaces were still operating, located in the area within the reach of the main walking and cycling trail and employed more than 10 people. At this stage, the many car sales companies in the area were excluded because based on few initial contacts their interest towards ACW promotion seemed mild. 

After accumulating the eligible workplaces, the management was contacted by e-mail or telephone to inquire their preliminary interest to participate in the study. The management was also asked to complete an electronic questionnaire about its past and current actions to promote employees’ ACW. 

In the beginning of 2016 the recruitment of the workplaces was expanded to Area 2 to increase the statistical power for Phase 2, which aimed to assess the additional effects of workplace-specific social and behavioral strategies on employees’ ACW. Another area was needed because all the voluntary workplaces from Area 1 were already participating in the study. Area 2 was selected because it had similar infrastructural features in terms of walking and cycling as Area 1 had after the trail improvements. In Area 2 the search for workplaces was conducted from the Internet and by visiting the area. The final checking about the minimum number of 10 employees was made by telephone. 

#### 2.2.2. Employees

The management determined the extent of each workplace’s participation. In small workplaces all employees were included but in larger workplaces the participation was limited to one or two units or departments to minimize the workload of the contact person in each workplace. The contact persons shared the names and e-mail addresses of the included employees with the researcher, who then was able to deliver personal study invitations to the employees. In some workplaces the personal invitations were delivered via contact person due to data privacy policy. A kick-off meeting was organized in all participating workplaces to introduce the study to the employees. 

### 2.3. Intervention

#### 2.3.1. Framework

**Figure 2:**
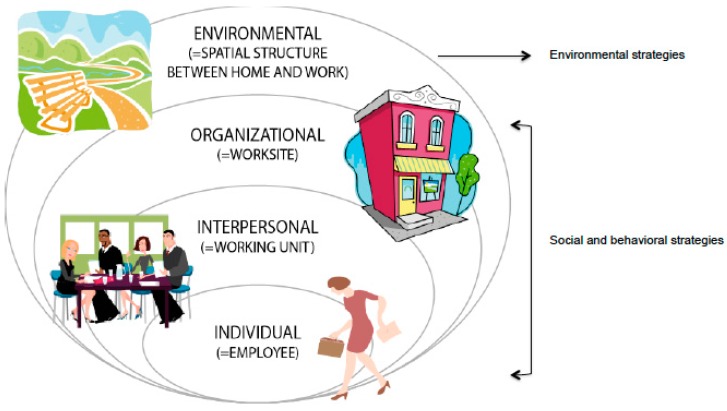
The socio-ecological approach of the intervention.

The intervention was based on the SE approach [25,26,27,28] (Figure 2), which integrates multiple life contexts relevant to behavior change [26]. SE models offer different pathways and levels to reach population groups, which may be underrepresented or unconnected with single-level interventions. For example, some people may be likely to engage in ACW through individual counseling while others are more dependent on social or environmental opportunities. 

#### 2.3.2. Environmental Strategies

**Figure 3:**
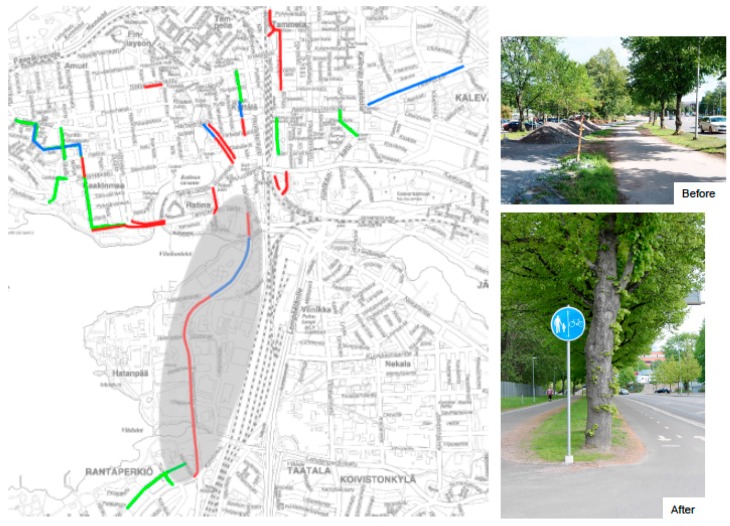
Environmental strategies related to Area 1. The improvements to the walking and cycling trails in 2014, 2015 and 2016 are marked with green, blue and red lines, respectively. Photographs on the right side illustrate the improvements on the main trail, which is shadowed in grey on the left.

In Phase 1 the effects of the improvements for the existing walking and cycling trails connected to the Area 1 will be assessed. The improvements of interest are the ones made by the City of Tampere with the regional funding of land use, housing and transportation (LHT network, www.mal-verkosto.fi/in_english) in 2014, 2015 and 2016. The locations and one example of the improvements are shown in Figure 3. 

The effects are examined by analyzing the changes in ACW from baseline (M1) to first follow-up (M2) in the whole sample of employees working in the workplaces in Area 1 (Figure 1). 

#### 2.3.3. Social and Behavioral Strategies

In Phase 2 the workplaces in Area 1 and Area 2 were separately arranged into pairs according to the number, sex distribution and educational level of the employees’ as well as to the proportion of employees’, who had a possibility to walk or cycle to work but did not report to do so in the baseline questionnaire. These workplace pairs were then randomly assigned into either experimental (EXP) or comparison group (COM). As a result of randomization, eight workplaces (6 from Area 1, 2 from Area 2) were allocated into EXP and eight (5 from Area 1 and 3 from Area 2) workplaces into COM (Figure 1). 

**Figure 4:**
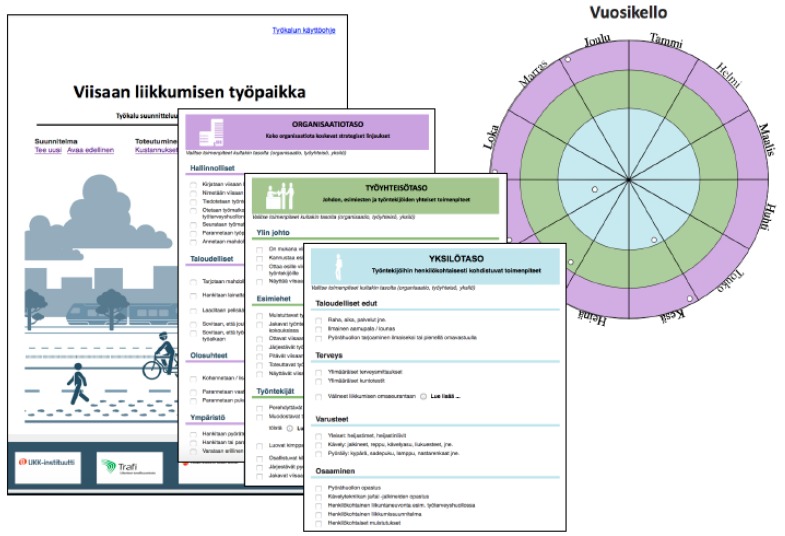
A workbook developed for the study to help the teams in the intervention workplaces to plan and implement social and behavioral strategies to promote active work commuting. The strategies were categorized into organizational (violet), working unit (green) and individual (light blue) level. The teams were to transfer the strategies chosen to a 1-year time plate (“Vuosikello” in the figure), which shows the number and timing of the strategies per level.

Each workplace in EXP nominated a team to plan and carry out social and behavioral strategies to promote ACW. The team selected the strategies most suitable for their workplace by utilizing a workbook, which was generated for the study and has since been modified into electronic format and expanded more widely to sustainable mobility (Figure 4). In the workbook the selection of strategies (n = 43) was categorized into organizational (n = 24), working unit (n = 9) and individual (n = 10) level. The teams were obliged to select at least one strategy from each level and to make an implementation plan for each strategy. 

**Figure 5:**
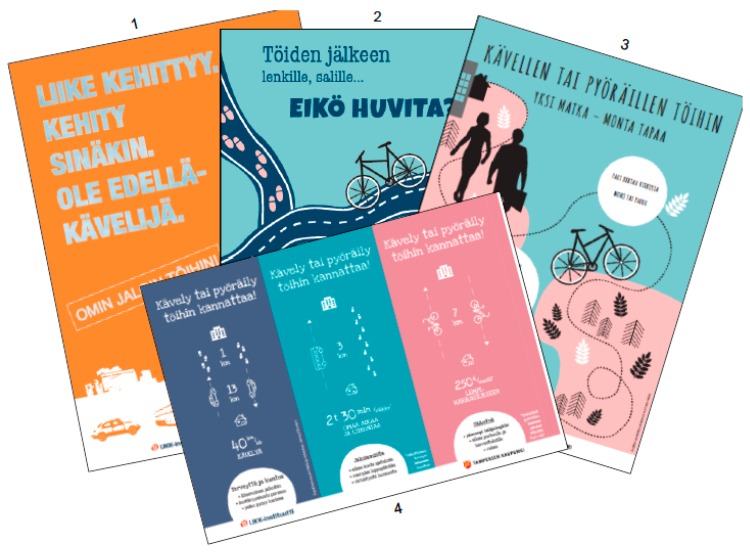
Posters (**1**–**3**) and a table triangle (**4**) developed for the study to provide supportive material for the workplaces in EXP. English translations for the main visible texts: Poster **1**: “Movement develops. Get involved. Be a forewalker”. Poster **2**: Going for a jog or to gym after work… Not in the mood? Poster **3**: Walking or cycling to work. One journey—many ways. Table triangle: It pays off to walk or cycle to work. Health and fitness… Strength for the day... Saving money....

The teams met one (JT) or two of the researchers (JT and OV) altogether three times for making the implementation plan. In the first meeting the teams were given the workbook, examples and supportive material (Figure 5), in the second meeting they were assisted to finalize the selection of strategies and in the third meeting the teams were helped to agree on practical arrangements such as timing and responsibilities related to the implementation. This procedure has been feasible in a previous workplace study aiming to promote physical activity and reduce sedentary behavior [31]. 

The workplaces in COM participated only in data collection but were offered the possibility to get the same support for promoting ACW after the study. 

### 2.4. Evaluation

The study includes process, impact, and efficiency evaluation (Table 1). In addition, environmental determinants and types that promote ACW and mediate and moderate the effects of environmental strategies are examined. 

#### 2.4.1. Process Evaluation

Process evaluation will be used to assess the external validity of the results such as the compliance of the workplaces and employees to the study, delivery of the intervention strategies and employees’ perceptions and awareness of the strategies. 

#### 2.4.2. Impact Evaluation

In Phase 1 the impact evaluation will show the effects of the environmental strategies in the dataset of Area 1 without any comparison between EXP and COM (Figure 1). For this purpose, the baseline measurements (M1) were completed in fall 2014 and repeated in fall 2016 (M2). Thus, the evaluation targets at environmental strategies, which were implemented after M1 and completed by M2. 

In Phase 2 the additional effects of workplace-specific social and behavioral strategies will be compared between EXP and COM in the combined datasets of Area 1 and Area 2. For this purpose, a new wave of M1 was carried out in the workplaces of Area 2 in spring 2016. In Area 1 the baseline measurements already conducted were utilized. The measurements were then repeated in both areas in spring 2017 (M3). 

The timing of the measurements was either fall or spring at all time points (M1, M2, M3) and the same in most workplaces throughout the study. In this respect, the measurements can be considered seasonally comparable in relation to walking and cycling conditions. 

##### Primary Indicator

The primary indicator of effects in both phases of the study is employees’ ACW (Table 1). Self-reported information on employees’ ACW was collected at all measurement points with a questionnaire, which the employees were able to complete either electronically or in paper format. The more specific indicators were employees’ primary mean of transportation to and from work, number of days per week actively commuting the whole journey to and from work, number of days per week walking part of the journey to or from work with information on kilometers and minutes and number of days per week bicycling part of the journey to or from work with information on kilometers and minutes. At baseline (M1) the questionnaire included also questions on employees’ background characteristics such as sex, age, height, weight, education, self-rated health, type of work, work ability, days absent from work, recovery from work and musculoskeletal symptoms. 

Objective data on ACW was collected with a light hip-worn triaxial accelerometer (Hookie AM20, Traxmeet Ltd., Espoo, Finland, www.traxmeet.com). The device has been found as valid as the most commonly used accelerometer (Actigraph GTX3, Actigraph LLC, Pensacola, FL, USA, www.theActigraph.com) in assessing adults’ physical activity and sedentary behavior [32] and the intensity of locomotion [33]. The accelerometers were delivered to the contact persons of the workplaces in the envelopes addressed separately to each employee. The envelope contained also written instructions to wear the accelerometer on the right hip during the waking hours for two to seven consecutive days and to remove the device only for sauna, shower and water activities. 

The acceleration data were collected in raw mode, which presents the acceleration data in actual G-force units (milligravity, mg) with a 100 Hz sampling frequency and a ±16,000 mg dynamic range. After the measurement period the stored data were transferred to a hard disk and analyzed in 6-s epochs. Non-wear time was defined as no movement detected in any epoch for at least 30 min. 

To facilitate the accelerometer use, the employees were offered a graphical feedback about their physical activity and sedentary behavior after each measurement point (M1, M2, M3). As another incentive, the employees had a possibility to use the accelerometer to perform a self-test for cardiovascular fitness and receive feedback from that. During the self-test the employee walked 6 min as fast as possible on a flat surface while using the activity monitor. The walking distance can be estimated from the acceleration signal and used as a proxy of fitness. Further, the locomotion-induced mean acceleration deviation is strongly (r > 0.9) associated with the incident heart rate, oxygen intake and energy consumption (MET) [33] allowing not only a reasonable estimate of submaximal and maximal performance but also energy consumption. 

Travel diaries were used to extract the information on ACW from the collected accelerometer data. On each accelerometer day the employees were asked to enter the following information to the day-specific row: date, working day/non-working day/teleworking day, time leaving home and arriving at work (h:min), all means of transportation used and separated with a comma (1 = walking, 2 = cycling, 3 = other active mode, 4 = car, 5 = bus, 6 = train, 7 = moped or motorcycle, 8 = other motorized mode), time starting and finishing work (h:min), time leaving work and arriving at home (h:min), and all means of transportation used and separated with a comma. The accelerometer and diary data were merged in Excel by using the data collection dates. The data on daily ACW was separated from the overall daily accelerometer data by using the times (h:min), which the employees had entered each day to the diary for going to work and returning from work. Entries on ACW from at least two working days were considered sufficient to describe employees’ daily ACW. 

##### Secondary Indicators

Information on employees’ self-rated health was collected at all measurement points in both areas to examine the change after each phase of the study and between the study groups. A single question was used, which has proved reliable in test-retest analysis [34,35] and shown to indicate physical and mental health in 19 countries including Finland [36] and to predict mortality in a randomly selected sample of Finnish population [37]. 

Subjective wellbeing at work was assessed at all measurement points in both areas to examine the change after each study phase and between the study groups. It included four single-item questions from the work ability index (WAI) [38,39]: current work ability compared with lifetime best (1–10, 0 = unable to work); work ability in relation to job demands (physical and mental, 5 categories each), own prognosis of work ability two years from now (3 categories) and number of days on sickness absence (5 categories). They have all been shown to perform well compared with the full WAI in predicting sickness absence in office workers [40]. Also a short version of the question on recovery from work (5 categories) was included, which has been shown to correlate with fatigue at work and work engagement [41]. 

Restrictions to ACW were assessed at M1 in both areas to discover their overall prevalence and at M2 in Area 1 to examine the change after environmental strategies. The information was obtained with a question “To what extent do the following factors restrict or prevent your active work commuting?” followed by a list of 42 potential factors grouped into environmental, organizational, work and personal level. An open space was provided after each group to allow respondents to express other possible restrictions. The response alternatives for each factor were “not at all”, “somewhat” and “completely”. 

Motivation for ACW was assessed at all measurement points in both areas to assess the change after each study phase and between the study groups. The motivation included three questions: employees’ willingness to increase ACW (walking and cycling asked separately; response alternatives “Yes/No, why?”), opportunities to actively commute at least part of the work journey (walking and cycling asked separately; response alternatives “Yes/No”) and intention to actively commute at least part of the work journey in the following week (walking and cycling asked separately; 0–5 days). 

The number of injuries due to ACW were self-reported by the employees at all measurement points in both areas to evaluate the change after each study phase and between the study groups. The question was “Have you had any injury or injuries due to active work commuting during the past month?” with the response alternatives “Yes, what?/No”. 

In Area 1, exclusively, the use of the main walking and cycling trail was examined at all measurement points to assess the change after each study phase. The indicator was the number of pedestrians and cyclists trespassing the trail during the afternoon peak hour measured by the year-round automated fixed-point calculations, which have been conducted in the area for more than ten years. New counting points for year-round monitoring of cyclists and pedestrians were executed for the study purposes. For example, an eco-counter automatic calculator was placed at a bridge, which captures the number of pedestrians and cyclists from both directions. 

In addition, the quality of the main walking and cycling trail going through Area 1 was assessed with an audit method at M1 and M2 to assess the change after environmental strategies. The same method was used in Area 2 at M1 to objectively ground-truth the comparability of the main walking and cycling trail to Area 1. The quality factors were chosen based on the previous literature on bicycle-friendly network [42,43,44]: average cycling speed (km/h), comfort of cycling (m/s^2^) and the rate of separation between the cycling and walking trail (%). The observed trails were cycled with a GPS tracker, which collected data on speed and location every second [45]. It also collected data on vertical acceleration to detect the smoothness of the road pavement representing comfort of cycling [46]. Rate of separation was determined by calculating the proportion of separated trail from a mixed trail. 

#### 2.4.3. Efficiency Evaluation

Efficiency evaluation produces information on the relationship between the intervention costs and effectiveness as well as on cost savings (Table 1). Cost-effectiveness analysis (Ex post) of the two phases of the study is performed in relation to the primary indicator, which is the employees’ ACW. The costs include direct, indirect and other costs documented during and after the study by the researchers, workplaces and the City of Tampere. Costs-to-effectiveness ratios are calculated for both phases. 

Health Economic Assessment Tool for Cycling and Walking (HEAT, www.euro.who.int/HEAT) is used to assess the cost savings of the intervention. HEAT is an online resource to estimate the economic savings resulting from reductions in mortality as a consequence of regular cycling or walking. It is based on best available evidence with parameters that can be adapted to fit specifically to walking or cycling and to various countries including Finland. HEAT calculates the answer to the following question: if x people cycle or walk y distance on most days, what is the economic value of mortality rate improvements? HEAT is used after the implementation of both phases of the study. 

#### 2.4.4. Environmental Evaluation

Information on different variables of physical environment considering land use, built environment as well as plot and street patterns [47] were collected at M1 in both areas to examine the inter-relationships between the variables [48] and to find urban environmental determinants that promote ACW. Another objective was to see, which variables of the built environment mediate and moderate the effects of environmental strategies on ACW. The assessment was based on GIS-analysis, which connects the information on physical environment to the routes, which employees were using for ACW. To get the information on actual routes, the employees were offered a possibility to download a smartphone application (http://www.sports-tracker.com) and share the information with the researcher (S-MS) or to use a map-based questionnaire (https://maptionnaire.com/en/) linked to the electronic questionnaire (only at M2). The routes from homes to workplaces were also calculated as the shortest routes between the start and end points of the actual routes. The built environmental features along the actual and shortest routes were then compared to find the preferred and avoided characteristics of the ACW routes. 

### 2.5. Statistics

Power calculations were based on *t*-test for difference of two independent means in a cluster-randomized design. A sample size of eight individual workplaces per group with 64 participants per workplace achieves 80% power to detect a group-wise difference of one in the change of number of daily walking or cycling sessions to work per week when the standard deviation is 3.0 and the intra-cluster correlation is 0.05. Generalized linear mixed models (SPSS Statistics for Windows, Version 24.0, IBM Corp., Armonk, NY, USA) will be used to analyze the effects of the intervention. 

## 3. Results

In Area 1 the search resulted altogether 76 workplaces of various commercial and industrial activities, such as health care, paper industry, heavy machinery and information technology. Of them 37 (48.7%) were eligible for the study based the manual, visual and telephone checking procedures. From Area 2 seven eligible workplaces were detected. 

After the inquiries to the management about their preliminary interest, eleven out of 27 (29.7%) workplaces from Area 1 and five out of seven (71.4%) workplaces from Area 2 agreed to participate in the study (Table 2). In Area 1 one half (49.9%) of the employees completed the baseline questionnaire and more than one third (39.7%) agreed to use the accelerometer and travel diary. In Area 2, the corresponding rates were 27.8% and 23.5%. 

The individual characteristics of the employees responding to the baseline questionnaire are described in Table 3. Approximately 40 percent of the respondents in both areas belonged to the age group of 30–45 years and both sexes were equally represented. Almost all had undergone some education after secondary or high school although the proportion of respondents having a university degree was greater in Area 2 than Area 1. In both areas majority of the respondents did sedentary work in regular day shift. Being overweight (BMI > 25) was somewhat more common in Area 1 than in Area 2 (49.1% vs. 40.8%). 

The baseline information on the primary indicator of impact evaluation (employees’ ACW) is provided in Table 4. In both areas the average distance from workplace to home was over ten kilometers and most respondents reported using a car to travel to and from work. The average number of days, which the respondents walked or cycled to or from work, was only around one day per week. Also, the kilometers and minutes walked to one direction were less than one kilometer and less than 10 min, respectively. In cycling the corresponding numbers were less than three kilometers and ten minutes. In both areas the accelerometer data combining walking and cycling showed a little more minutes for active commuting to work (11.6 and 11.3) and clearly more minutes for returning home (26.3 and 25.0). However, the data is still incomplete and the minutes are likely to change as walking and cycling are separated. 

The baseline information on the secondary indicators of the impact evaluation is provided in Table 5. Three thirds (75.6%) in Area 1 and even more of the respondents (79.6%) in Area 2 rated their health fairly good or good at baseline. Also, based on the sub-indicators, their subjective wellbeing at work seemed generally good. In both areas, the average number of restrictions to ACW was the highest at personal and second highest at organizational level. Overall, 40 to 70 percent of the respondents were willing to increase walking or cycling with a greater willingness for walking. More than one half of the respondents reported having an opportunity to either walk or cycle at least part of their work journey. However, only less than two percent had intention to do so in the following week. 

According to the traffic calculations notably more cyclist than pedestrians were observed at all counting points related to the main walking and cycling trail in Area 1. Also, the number of observations distributed more evenly in cyclists (from 134 to 186) than in pedestrians (from 34 to 133) at different counting points. Based on the three quality factors the two areas seemed to be quite comparable in terms of the main walking and cycling trail. 

## 4. Discussion

The study aims at examining the effects of socio-ecological approach in a sample of workplaces and employees exposed first to environmental and then to social and behavioral strategies to promote ACW. This paper describes the protocol, evaluation and baseline findings of the study. 

### 4.1. Study Strengths

The study utilizes socio-ecological framework in ACW promotion. As far as we know, similar approach has not been used in previous studies. It is also the first study in Finland carried out in collaboration with interdisciplinary group of researchers and practitioners in the field of transportation, urban design, physical activity and sustainable development. Presumably, this type of collaboration is increasingly needed in the future to change people’s transportation habits. 

The major strength of the study is its implementation in a real-world context: the environmental strategies were part of the city traffic plans and the social and behavioral strategies were integrated into the workplaces’ health promotion practices and implemented mostly by the workplaces themselves. Also, the intervention included various types of workplaces and the feasibility of the protocol related to the social and behavioral strategies had been previously piloted. All these things are likely to enhance the transferability of the results into the practice. 

A randomized controlled design was used in Phase 2, which is always laborious in natural experiments. However, it improves the possibility to separate the intervention effects from numerous other activities that inevitably take place over time in a complex socio-ecological system. For instance, there may be general change in public attitude or atmosphere towards ACW, which dilutes or strengthens target group’s behavior regardless of the intervention. The controlled design helps to see the intervention effects over these changes and thus enhances the reliability of the findings. 

The study includes process, impact, efficiency and environmental evaluation. This enables one to discover explanations to the findings, justify the need for disseminating the approach more widely and to connect the findings to infrastructural level. Also, the number and diversity of the primary and secondary indicators is high and their assessment is based on a wide variety of subjective and objective measures both improving the likelihood of revealing potential changes. 

### 4.2. Study Limitations

The workplaces were enrolled from two specific areas only, which may limit the generalization of the results to other types of urban environments. The voluntary basis of the workplaces may also have caused selection bias to the sample in such a way that only the most compliant workplaces participated in the study. As a result, the findings on social and behavioral strategies may best apply to workplaces, which are in a good position in relation to their financial and staff resources to promote employees’ ACW and health in general. 

The selection bias may exist also at the employee level [49]. This means that there is a risk that mainly the most compliant employees, who are usually healthier, participated in the study thus weakening the generalization of the findings to the original sample of employees. In this study, the average participation rate at baseline was in both areas above 33%, which is a median in worksite health promotion programs [49]. Moreover, the individual characteristics of the employees at baseline were similar to the general working-aged population in Finland [50]. Arranging the kick-off meeting for the staff and nominating a contact person in each workplace may have improved participation. It is nevertheless clear that the participation will drop at M2 and M3 challenging the generalizability of the findings and possibly also the statistical power of the study. 

The exposure especially to the environmental strategies prior to M2 may be too short for modifying employees’ travel behavior. However, the timeline for the intervention duration was pre-determined by the funders and could not have been extended. To compensate the briefness of the exposure and to see even the smallest possible changes at multiple levels, a wide variety of measures were used. Still, as seen from Table 5 some of the secondary indicators especially under “subjective wellbeing at work” show high values already at baseline, which makes it difficult to achieve or see any further improvements. 

### 4.3. Usability of the Findings

The findings on the process and effectiveness evaluation can be used to develop practices to promote ACW at environmental and workplace level. The information thus benefits especially agents promoting ACW and other means of sustainable mobility at practical level. Efficiency evaluation gives information to decision-makers about the economic value of promoting ACW with environmental and social and behavioral strategies. As the information accumulates through other studies, the decision-makers can better target their policy actions for ACW. Environmental findings benefit traffic planners and urban designers by providing them with novel information about the environmental aspects of ACW. Connecting the information to the findings of previous and future studies can help in designing workplace areas that promote ACW more efficiently. 

The findings of the study will be disseminated to HR managements, physical activity promoters and the agents of sustainable mobility through websites, journal articles, events and social media. In the end of 2017 a closing seminar will be arranged to the relevant stakeholders to discuss the findings and their translation into practice. The workbook developed for the study in order to help the workplaces to make their actions plans for ACW is currently being expanded to cover sustainable mobility more widely, transformed into electronic form and stored in a database, which is freely accessible to all the workplaces in Finland through registration. The data accumulating to the database enables decision-makers and developers of sustainable mobility to gain information on the most popular strategies selected by the workplaces as well as on their realization, costs and effects. 

## 5. Conclusions

The study aims at promoting ACW in two urban areas involving 16 workplaces. In Phase 1, environmental strategies were carried out in Area 1 to discover the effects of improvements to walking and cycling trails. In Phase 2, the workplaces in both areas were randomized to intervention and comparison group to see the effects of social and behavioral strategies carried out by the workplaces. Wide variety of indicators and measures were used to evaluate the process, effects and efficiency of the multilevel intervention as well as the environmental aspects of ACW. 

Both phases of the study have now been completed and the data is under analysis. The findings can be used to develop practices to promote ACW at environmental and workplace level and to give information to decision-makers about how to target their policy actions for ACW. 

## Figures and Tables

**Table 1:** Evaluation of the study.

Evaluation Questions/Indicators	Measurement Points ^1^			Measures
	M1	M2	M3	
**Process evaluation**				
What percentage of potentially eligible employees took part and how representative were they?	X	X	X	Employee questionnaire
What percentage of workplaces volunteered and how representative were they?	X	X	X	Documentation during the recruitment
				Management questionnaire
To what extent did the environmental strategies succeed as intended in Area 1?		X		Monitoring and supervision by the authorities of City of Tampere
To what extent did the employees in Area 1 pay attention to the environmental strategies?		X		Employee questionnaire
To what extent were the social and behavioral strategies planned and delivered as intended?			X	Researchers’ visits to the workplaces
				Workbooks completed by the workplaces
				Employee questionnaire
**Impact evaluation**				
*Primary indicator*				
number of active commuters to work	X	X	X	Employee questionnaire, accelerometer, travel diary
*Secondary indicators*				
self-rated health	X	X	X	Employee questionnaire
subjective wellbeing at work	X	X	X	
restrictions to active commuting to work	X	X		
motivation for active commuting to work	X	X	X	
injuries due to active commuting to work	X	X	X	
use of the main walking and cycling trail	X	X	X	Traffic calculations
quality of the main walking and cycling trail	X	X		Auditing: cycling with GPS
**Efficiency evaluation**				
effects of the strategies in relation to costs		X	X	Cost-effectiveness ratio
cost-savings of the intervention		X	X	Health Economic Assessment Tool for walking and cycling (HEAT)
**Environmental mediators and moderators**				
What urban environmental variables and types promote active commuting to work?	X	X		Employee questionnaire, Smartphone App, Map-based questionnaire
Which urban environmental variables and types mediate and moderate the effects of environmental strategies?		X		

^1^ M1 = baseline measurements; M2 = measurements after environmental strategies; M3 = measurements after social and behavioral strategies.

**Table 2:** Participating workplaces and their field of activity, number (n) of employees invited, respondents to the baseline questionnaire and employees using the accelerometer and diary at baseline.

Workplace	Field of Activity	Employees n (% of Area’s Subtotal)	Respondents n (% of Employees)	Accelerometer n (% of Employees)
Area 1					
1	Infrastructure	54 (3.0)	34 (63.0)	27 (50.0)
2	Engineering & consulting	44 (2.4)	27 (61.4)	18 (40.9)
3	Technology industries	359 (19.7)	206 (57.4)	54 (15.0)
4	Mobile networks	276 (15.1)	109 (39.5)	78 (28.3)
5	Public administration	323 (17.7)	204 (63.2)	101 (31.3)
6	Health care	138 (7.6)	97 (70.3)	100 (72.5)
7	Social services	171 (9.4)	56 (32.7)	63 (36.8)
8	Information technology	117 (6.4)	50 (42.7)	50 (42.7)
9	Forest industries	100 (5.5)	53 (53.0)	54 (54.0)
10	Telecommunication	213 (11.7)	60 (28.2)	61 (28.6)
11	Mobile software	28 (1.5)	14 (50.0)	19 (67.9)
Subtotal		1823 (100)	910 (49.9)	724 (39.7)
Area 2					
1	Technical research	251 (30.4)	70 (27.9)	43 (17.1)
2	Technology university	175 (21.2)	26 (14.9)	30 (17.1)
3	Vocational school	177 (21.4)	38 (21.5)	26 (14.7)
4	Software development	63 (7.6)	33 (52.4)	36 (57.1)
5	College	160 (19.4)	63 (39.4)	59 (36.9)
Subtotal		826 (100)	230 (27.8)	194 (23.5)
**Total**		**2652**	**1144 (43.1)**	**918 (34.6)**

**Table 3:** Individual characteristics of the employees at baseline in Area 1 and Area 2. Means and standard deviations (SD) or numbers (n) and proportions (%).

Baseline Characteristics of the Employees	Area 1 (n = 910)	Area 2 (n = 230)
Age in years, mean (SD)	44.5 (32.3)	44.1 (11.4)
Age-group, n (%)		
<30 years	111 (12.4)	32 (14.0)
30–45 years	397 (44.4)	90 (39.9)
46–55 years	221 (24.7)	60 (26.2)
>55 years	165 (18.5)	47 (20.5)
Women, n (%)	476 (52.7)	125 (54.3)
Married, n (%)	715 (79.2)	166 (72.8)
Caretaker to children under 18 years of age, n (%)	388 (43.7)	81 (35.8)
Education, n (%)		
Secondary school or high school graduate	57 (6.3)	10 (4.3)
Polytechnic or vocational school	455 (50.3)	70 (30.4)
University degree	388 (42.9)	150 (65.2)
Other	4 (0.4)	0
Working hours, n (%)		
Regular day work	800 (88.8)	200 (87.3)
Shift-work (2 or 3 shifts)	33 (3.7)	5 (2.2)
Irregular or other hours	11 (1.2)	7 (3.1)
Part-time job	34 (3.8)	11 (4.8)
Other	23 (2.6)	6 (2.6)
Type of work, n (%)		
Sedentary work	760 (84.1)	200 (87.0)
Mainly standing or light ambulatory work without carrying	79 (8.7)	22 (9.6)
Mainly ambulatory work with carrying or climbing stairs	49 (5.4)	7 (3.0)
Heavy or extremely heavy physical work	15 (1.7)	1 (0.4)
Body mass index (kg/m^2^), mean (SD)	25.6 (3.9)	25.1 (4.3)
Body mass index > 25, n (%)	439 (49.1)	91 (40.8)
Smoking; yes, n (%)	68 (7.6)	12 (5.3)

**Table 4:** Baseline information related to the primary indicator of the study (active commuting to work, ACW) in Area 1 and Area 2. Means and standard deviations (SD) or numbers (n) and proportions (%).

Baseline Information on the Primary Indicator	Area 1	Area 2
**Employee questionnaire**	**n = 909**	**n = 230**
Kilometers from home to work, mean (SD)	14.7 (17.3)	13.3 (20.8)
Primary mean of transportation *to* work, n (%)		
By car	545 (60.0)	118 (51.8)
By bus	123 (13.5)	26 (11.4)
By moped or motorcycle	3 (0.3)	2 (0.9)
By train	9 (1.0)	0
By foot	53 (5.8)	32 (14.0)
By bicycle	174 (19.1)	47 (20.6)
Other	2 (0.2)	3 (1.3)
Primary mean of transportation *from* work, n (%)		
By car	536 (59.2)	117 (51.1)
By bus	121 (13.4)	27 (11.8)
By moped or motorcycle	4 (0.4)	2 (0.9)
By train	9 (1.0)	0
By foot	58 (6.4)	34 (14.8)
By bicycle	176 (19.4)	47 (20.5)
Other	2 (0.2)	2 (0.9)
Number of days per week actively commuting the whole journey *to and from* work, mean (SD)		
Walking	0.4 (1.1)	0.8 (1.7)
Bicycling	1.0 (1.7)	1.0 (1.7)
Number of days per week *walking* part of the journey *to* work, mean (SD)	0.9 (1.7)	1.3 (2.0)
Kilometers walked, mean (SD)	0.8 (1.4)	0.6 (1.3)
Minutes walked, mean (SD)	7.5 (12.1)	5.8 (9.6)
Number of days per week *bicycling* part of the journey *to* work, mean (SD)	0.9 (1.7)	1.0 (1.7)
Kilometers bicycled, mean (SD)	2.5 (4.2)	2.1 (4.1)
Minutes bicycled, mean (SD)	8.8 (12.8)	7.3 (13.4)
Number of days per week *walking* part of the journey *from* work, mean (SD)	0.9 (1.7)	1.3 (2.0)
Kilometers walked, mean (SD)	0.8 (1.4)	0.7 (1.3)
Minutes walked, mean (SD)	8.2 (13.0)	6.1 (10.0)
Number of days per week *bicycling* part of the journey *from* work, mean (SD)	1.0 (1.8)	1.0 (1.7)
Kilometers bicycled, mean (SD)	2.5 (4.2)	2.1 (4.2)
Minutes bicycled, mean (SD)	9.4 (13.6)	7.2 (13.5)
**Accelerometer and travel diary ^1^**	**n = 240 (to)** **n = 267 (from)**	**n = 87 (to)** **n = 103 (from)**
Daily minutes of active commuting to work, mean (SD)	11.6 (9.7)	11.3 (9.6)
Daily minutes of active commuting from work, mean (SD)	26.3 (15.8)	25.0 (18.3)

^1^ Accelerometer data and diary entries on ACW from at least two working days. Mean number of monitoring days for both directions (to/from) was 4.0. The number of employees with valid data is not yet complete. In future analysis walking and cycling will be separated and the intensity of ACW will be specified.

**Table 5:** Baseline information related to the secondary indicators of the study in Area 1 and Area 2. Means and standard deviations (SD) or numbers (n), proportions (%) or other suitable units (auditing).

Baseline Information on the Secondary Indicators	Area 1		Area 2
**Employee questionnaire**	**n = 909**		**n = 230**
Self-rated health; fairly good or good, n (%)	678 (75.6)		183 (79.6)
Subjective wellbeing at work			
Current work ability compared with lifetime best; 1–10, mean (SD)	8.6 (1.1)		8.5 (1.2)
Work ability in relation to physical job demands; fairly or very good, n (%)	769 (85.0)		202 (88.2)
Work ability in relation to mental job demands, fairly or very good, n (%)	774 (85.6)		190 (83.3)
Own prognosis of work ability two years from now; fairly sure, n (%)	856 (94.8)		212 (92.6)
Number of days on sickness absence during the past year; more than 6 days, n (%)	63 (7.0)		12 (5.2)
Recovery from work after a working day or shift; well or fairly well, n (%)	698 (77.3)		170 (74.9)
Number of factors restricting or preventing active commuting to work somewhat or completely, mean (SD)			
Environmental	3.4 (2.5)		3.2 (2.6)
Organizational	1.7 (1.9)		1.1 (1.7)
Work	2.0 (1.9)		2.5 (2.1)
Personal	5.4 (3.6)		5.5 (3.7)
Motivation for active commuting to work			
willingness to increase *walking*; yes, n (%)	352 (43.2)		91 (49.5)
willingness to increase *bicycling*; yes, n (%)	528 (61.8)		137 (71.7)
opportunity to *walk* at least part of the work journey; yes, n (%)	540 (61.5)		116 (59.5)
opportunity to *bicycle* at least part of the work journey; yes, n (%)	567 (65.2)		126 (64.3)
intention to *walk* least part of the work journey in the following week, mean number of days (SD)	1.4 (1.8)		2.1 (1.9)
intention to *bicycle* at least part of the work journey in the following week, mean number of days (SD)	1.7 (1.9)		2.0 (1.7)
Number of employees reporting injuries due to active work commuting, mean (SD)	18 (2.0)		8 (3.6)
**Traffic calculations (Area 1 only)**			
Number of pedestrians (Ped) and cyclists (Cyc) trespassing the main walking and cycling trail during the afternoon peak hour	Ped	Cyc	-
Counting point 1	78	186	-
Counting point 2	34	160	-
Counting point 3	133	166	-
Counting point 4	64	134	-
**Auditing**			
Quality of the main walking and cycling trail ^1^			
Average speed of cycling (km/h)	14.3		14.8
Comfort of cycling (m/s^2^)	0.97		0.85
Rate of separation (%)	0.07		0.15

^1^ Performed in Area 2 only to objectively ground-truth the comparability of the main walking and cycling trail to Area 1.
